# Coral-like 3D porous Co–BDC nanostructures synthesized by simple one-step chronoamperometry for electrochemical detection of Cd^2+^

**DOI:** 10.1039/d6ra02923j

**Published:** 2026-07-20

**Authors:** Dinh Dung Luong, Thi Hai Yen Pham, Tien Dat Doan, Nhung Hac Thi, Ho Thi Oanh, Hong Tham Nguyen, Thi Thu Ha Vu, Tuyen Van Nguyen, Bui Dinh Tu, Thi Kim Dung Hoang, Mai Ha Hoang

**Affiliations:** a Institute of Chemistry, Vietnam Academy of Science and Technology 18 Hoang Quoc Viet, Nghia Do Ward Hanoi 10000 Vietnam hoangmaiha@ich.vast.vn; b Faculty of Engineering Physics and Nanotechnology, VNU University of Engineering and Technology, Vietnam National University 144 Xuan Thuy, Cau Giay Hanoi 10000 Vietnam; c Institute of Advanced Technology, Vietnam Academy of Science and Technology No. 1B, TL29 Str., An Phu Dong Ward Ho Chi Minh City 70000 Vietnam

## Abstract

Metal–organic frameworks (MOFs) have emerged as promising materials for the electrochemical sensing of toxic heavy metals such as Cd^2+^, owing to their high surface area and tunable porosity. Herein, a porous coral-like 3D Co–BDC MOF film was rapidly synthesized on a glassy carbon electrode (GCE) *via* a simple one-step cathodic chronoamperometry method. Under optimized conditions (−1.4 V for 300 s, Co^2+^ : BDC ratio of 1 : 2), a uniform and interconnected porous nanostructure was obtained that effectively facilitates Cd^2+^ diffusion and preconcentration. The Co–BDC/GCE exhibited a 15.3-fold enhancement in square wave anodic stripping voltammetry (SWASV) signal compared with the bare GCE. A wide linear response was achieved from 0.05 to 30 ppb (*R*^2^ = 0.9995) with a low limit of detection (LOD) of 0.035 ppb. The sensor also demonstrated excellent reproducibility (RSD 1.35%), high selectivity against common interferents, and acceptable long-term stability over 50 days. Its practical applicability was successfully validated through accurate quantification of Cd^2+^ in real water (lake and river) and vegetable samples, with all detected concentrations well below the regulatory limits set by the World Health Organization (WHO) and European Union (EU). This binder-free, scalable approach highlights the potential of electrochemically deposited Co–BDC for sensitive environmental and food safety monitoring of trace Cd^2+^.

## Abstract 2

One-step electrodeposited Co–BDC on GCE enables ultrasensitive SWASV detection of Cd^2+^ with a low detection limit of 0.035 ppb and good performance in real water samples.

## Graphical abstract

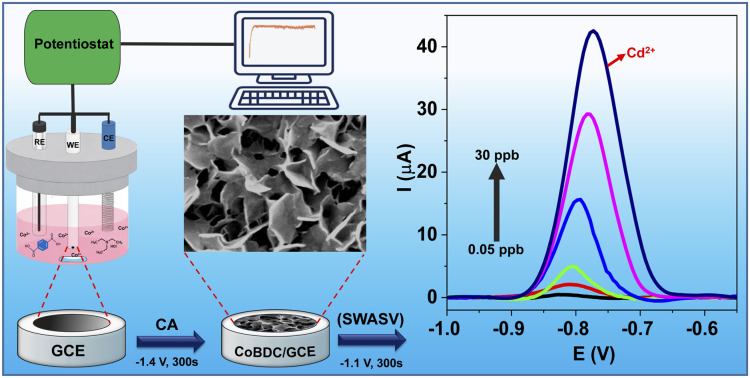

## 1. Introduction

Metal–organic frameworks (MOFs) are highly promising materials for electrochemical sensing applications owing to the synergistic integration of metal nodes and organic ligands, which yields properties superior to those of their individual components. The high surface area and intrinsic porosity of MOFs provide abundant active sites, facilitating efficient analyte adsorption and preconcentration.^1^ Furthermore, their porous frameworks can be precisely tailored to enhance both selectivity and binding affinity toward target analytes.^2^ Additionally, the presence of metal sites with Lewis acidity and redox capabilities facilitates electron transfer and these serve as electrocatalytic centers.

Since 2014, MOF-based electrochemical sensing has advanced significantly, particularly for the detection of heavy metal ions such as Cd^2+^. Commonly used MOFs for Cd^2+^ sensing include Zr^−^ and Fe-based frameworks,^3–6^ as well as those derived from Zn,^7^ Co,^8^ Yb,^9,10^ and Cr.^11^ Most studies employ carboxyl- and amino-functionalized benzene-based linkers, with 2-aminoterephthalic acid being the most prevalent. Notable examples include NH_2_-MIL-88(Fe)/rGO composites for the sensitive and selective detection of Cd^2+^, Pb^2+^, and Cu^2+^;^6^ ZnBDC–NH_2_/GO/l-cysteine/gold nanoparticles for enhanced Cd^2+^ and Pb^2+^ sensing;^7^ and ZIF-67/rGO for Cd^2+^ and Pb^2+^ detection.^8^ These composites leverage large surface areas, strong adsorption capacity, and improved conductivity to boost performance.

Recent research has focused on the development of scalable, accurate, simple, energy-efficient, and miniaturized MOF-based sensors.^12–15^ While conventional synthesis methods (hydrothermal, microwave-assisted, mechanochemical, ultrasonic and slow evaporation) remain widely used, electrochemical deposition has gained considerable attention as a binder-free, *in situ* technique for directly coating MOF films on conductive substrates, thereby improving device performance by eliminating polymeric binders.^16–20^ Two main approaches exist: anodic electrochemical deposition (AED) and cathodic electrochemical deposition (CED).^18^ AED requires sacrificial metal electrodes, making it unsuitable for inert substrates such as glassy carbon, gold, platinum, or ITO/FTO glass. In contrast, CED, in which cathodic reduction generates a local basic environment for ligand deprotonation and MOF self-assembly, is more versatile, offering excellent controllability, rapid synthesis, high surface area, and uniform film thickness.^21–24^ Despite these advantages, the application of electrochemically synthesized MOFs for sensor development remains in its early stages.

Consequently, electrodeposited MOFs have been widely explored for applications in electrocatalysis, energy storage, gas separation, and electrochromic devices. However, their application in electrochemical sensing has primarily focused on gases, glucose, and biomolecules.^25,26^ In contrast, the direct use of single-component electrodeposited MOF films as stripping voltammetric sensing interfaces for heavy-metal determination remains largely unexplored. Most reported MOF-based heavy-metal sensors rely on chemically synthesized MOFs, which must be subsequently combined with conductive materials such as graphene, carbon nanotubes, conductive polymers, or metal nanoparticles to improve electrical conductivity and sensing performance.^27–29^ This fabrication strategy generally involves multiple preparation steps and the use of additional functional components. Therefore, developing a directly electrodeposited single-component MOF film as a stripping-voltammetric sensing interface represents an attractive and simplified strategy for Cd^2+^ determination.

In this work, a directly electrodeposited single-component Co–BDC sensing interface was developed for the sensitive determination of Cd^2+^. A porous Co–BDC film was grown *in situ* on a glassy carbon electrode through a one-step cathodic electrodeposition process without the use of pre-synthesized MOFs, conductive nanomaterials, or polymeric binders. The resulting interconnected architecture facilitates efficient Cd^2+^ diffusion and preconcentration, enabling highly sensitive detection by square-wave anodic stripping voltammetry. Furthermore, the sensing mechanism was elucidated through electrochemical characterization combined with FTIR and EDX analyses after Cd^2+^ adsorption, providing experimental evidence for the interaction between Cd^2+^ and the Co–BDC framework. The practical applicability of the proposed sensor was further validated by the accurate determination of Cd^2+^ in real water and vegetable samples.

## 2. Experimental

### 2.1. Materials

Cobalt(ii) nitrate hexahydrate (Co(NO_3_)_2_·6H_2_O, 99%), terephthalic acid (H_2_BDC, 98%), triethylammonium hydrochloride (Et_3_N·HCl, 99%), potassium ferricyanide (K_3_[Fe(CN)_6_], 99%), potassium ferrocyanide trihydrate (K_4_[Fe(CN)_6_]·3H_2_O, 98%), monopotassium phosphate (KH_2_PO_4_, 99%), dipotassium phosphate (K_2_HPO_4_, 98%), phosphoric acid (H_3_PO_4_, 85%), acetic acid (CH_3_COOH, 99%), sodium acetate (CH_3_COONa, 99%), potassium chloride (KCl, 99.5%), hydrochloric acid (HCl, 37%), potassium hydroxide (KOH, 85%), and *N*,*N*-dimethylformamide (DMF) were purchased from Sigma-Aldrich. The Cd^2+^ standard solution (1000 ppm) was supplied by Fisher Scientific. Double-distilled water, ethanol, and acetone were used for solution preparation and cleaning.

### 2.2. Apparatus

All electrochemical measurements were carried out using a potentiostat/galvanostat (Autolab PGSTAT302N, Metrohm) with a three-electrode system, consisting of an Ag/AgCl (3 M KCl) reference electrode, a platinum wire counter electrode, and the working electrode. The electrochemical properties of the MOF-modified electrode were examined in a 5 mM [Fe(CN)_6_]^3−/4−^ solution using cyclic voltammetry (CV) in the potential range from 0.6 V to −0.2 V, and electrochemical impedance spectroscopy (EIS) in the frequency range from 10^5^ Hz to 0.01 Hz, with the initial potential set at the open-circuit potential. The electrochemically active surface area (ECSA) of the bare GCE and Co–BDC/GCE electrodes was estimated from the double-layer capacitance (*C*_dl_). Cyclic voltammetry measurements were carried out in 0.1 M NaNO_3_ within a non-faradaic potential window of 0.05–0.20 V at scan rates of 20, 40, 60, 80, and 100 mV s^−1^.

Other physical and chemical properties of the MOFs were studied using X-ray diffraction (XRD, Bruker D8 Advance, Germany), attenuated total reflection-Fourier transform infrared spectroscopy (ATR-FTIR, PerkinElmer L1600400 Spectrum TWO, UK), field emission scanning electron microscopy (FESEM, JSM-IT800/JEOL, Japan), and energy-dispersive X-ray spectroscopy (EDX) integrated with SEM using an Ultim Max 65 detector (Oxford Instruments, UK). The surface porosity of the electrodeposited Co–BDC film was quantified from FESEM images using Fiji (ImageJ, version 1.54g). FESEM images recorded at 5000× and 20 000× magnifications were thresholded and converted into binary images. The surface porosity was then calculated using the area fraction function in Fiji (ImageJ).

### 2.3. Synthesis of MOFs

A Co–BDC MOF layer was directly synthesized on the surface of GCE *via* cathodic electrochemical deposition.

Prior to MOF deposition, the GCE was polished with 5000-grit sandpaper and cleaned with ethanol, followed by rinsing with double-distilled water and drying at room temperature. Subsequently, a three-electrode system was immersed in a DMF solution containing the precursors: 10 mM Co(NO_3_)_2_, 20 mM H_2_BDC, and 1 mM Et_3_N·HCl. A constant potential of −1.4 V was applied for 300 s under magnetic stirring at 150 rpm to grow the MOF material on the GCE surface. The resulting electrode was denoted as Co–BDC/GCE.

The synthesis conditions for MOF formation, including precursor concentration ratios (from 2 : 1 to 1 : 5), applied potentials (−1.2 V, −1.4 V, and −1.6 V), and deposition times (120–360 s), were systematically varied and optimized. The optimal conditions (Co^2+^ : BDC ratio of 1 : 2, applied potential of −1.4 V, and deposition time of 300 s) were selected based on the highest SWASV response of Cd^2+^.

### 2.4. Cd^2+^ analysis

Cd^2+^ was analyzed using square wave anodic stripping voltammetry (SWASV), with baseline-corrected peak currents taken as the analytical signals. The analytical conditions, including the electrolyte composition, pH, deposition potential, and enrichment (accumulation) time, were investigated and optimized prior to establishing the calibration curve.

### 2.5. Pretreatment of real samples

The water samples were collected from West Lake in Ha Noi and the Cau River in Thai Nguyen Province. The sweet potato vegetable was purchased from a local market. Before analysis, the water samples were filtered through 10 µm glass fiber filter paper to remove suspended solids.

The vegetable samples, including both leaves and stems, were thoroughly rinsed with double-distilled water to remove dirt, soil, and other visible contaminants. The samples were subsequently air-dried under gentle, controlled airflow to prevent compositional changes and then accurately weighed. They were then oven-dried at 100 °C until a constant weight was obtained. The dried samples were finally ground into a fine powder for further analysis. A 4.412 g portion of the powdered sample was digested in 300 mL of concentrated HNO_3_. The digested solution was analyzed using the standard addition method with the Co–BDC/GCE electrode and validated by inductively coupled plasma mass spectrometry (ICP-MS).

Cd signal measurements were performed in triplicate to ensure the reliability of the analytical results.

All experiments were conducted at room temperature.

## 3. Results and discussion

### 3.1. MOFs electrode characteristics

To elucidate the electrochemical processes occurring in the precursor solution, cyclic voltammetry (CV) measurements were performed within a potential window from 1.0 to −1.6 V (Fig. S1a).

While no peak currents were observed in the pure DMF solution, the solution containing Et_3_N·HCl (red curve) exhibited a gradual increase in cathodic current between −0.4 and −1.2 V. This behavior is likely associated with the reduction-induced deprotonation of Et_3_N·HCl.^30^

Upon the addition of Co(NO_3_)_2_ and H_2_BDC to the electrolyte solution, distinct cathodic peaks emerged at −0.8 and −1.3 V. These peaks are attributed to the electrochemical reduction of NO^3−^ to NO^2−^, which is accompanied by the simultaneous generation of OH^−^ (reactions (1) and (2)), thereby facilitating MOF assembly (reactions (3) and (4)).^23^ Consequently, a deposition potential more negative than −1.2 V was selected to drive the MOF growth. The proposed pathway for the electrochemical synthesis of Co–BDC proceeds through the following reactions.

1 NO_3_^−^ + 2H^+^ + 4e → NO_2_^−^ + H_2_O

2 NO_2_^−^ + 3H_2_O ↔ NO_3_^−^ + 2OH^−^ + 2H_2_

3 H_2_BDC + OH^−^ ↔ HBDC^−^ + H_2_O

4 HBDC^−^ + Co^2+^ ↔ Co–BDC (MOF)

**Scheme 1:**
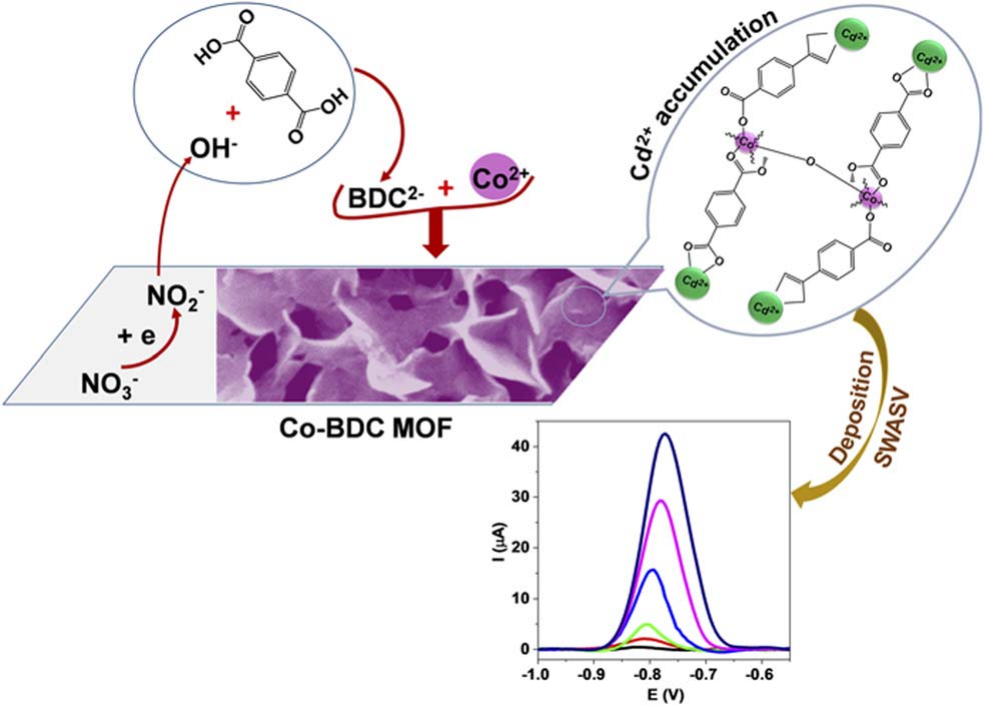
Illustrates the electrochemical formation of the Co–BDC film on the GCE and the proposed mechanism for Cd^2+^ sensing.

**Fig. 1:**
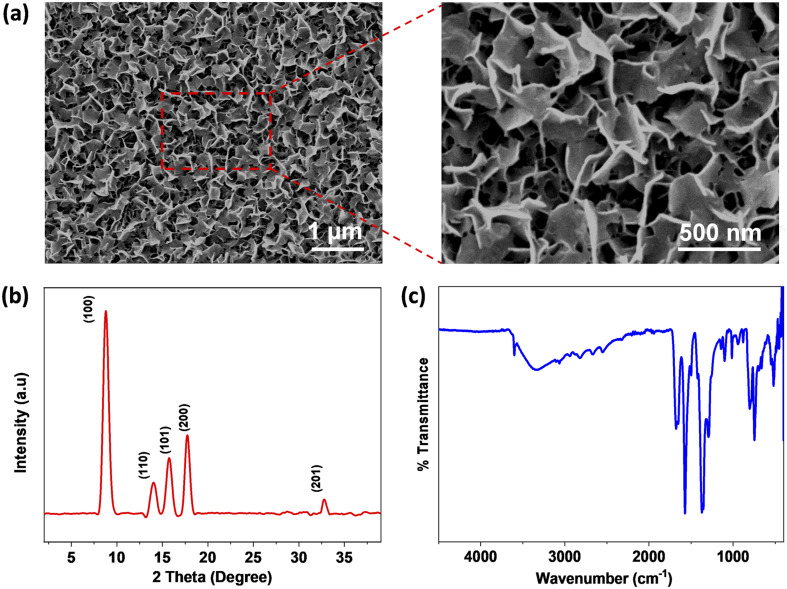
(a) FESEM images at different magnifications and (b) XRD pattern of the Co–BDC/GCE film synthesized at −1.4 V for 300 s; (c) ATR-FTIR spectrum of the Co–BDC/GCE film synthesized at −1.4 V for 1200 s.

Compared with the electrode modified by drop-casting of chemically synthesized Co–BDC, the electrochemically prepared counterpart features multidirectionally interconnected nanosheets that form a more uniform and highly porous network across the entire surface. Scheme 1 illustrates the electrochemical formation of the Co–BDC film and the proposed mechanism for Cd^2+^ sensing. The electrochemical deposition of Co–BDC onto the GCE was carried out at −1.4 V for 300 s (Fig. S1b), yielding a uniform layer across the electrode surface. SEM characterization (Fig. 1a) reveals a highly porous, coral-like interconnected framework composed of thin, wavy nanosheets oriented in multiple directions, indicative of a well-developed three-dimensional architecture.^31,32^ To provide a quantitative evaluation of this morphology, image analysis was performed using Fiji (ImageJ) based on approximately 1500 pores identified from a high-magnification (50 000×) FESEM image (Fig. S2). The analysis revealed that the framework consists of nanosheets with an apparent thickness of 15.5 ± 7.2 nm and interconnected pores with characteristic dimensions of 47.6 ± 30.6 nm (major axis) and 28.1 ± 20.0 nm (minor axis), confirming the formation of a well-defined nanostructured porous network. Furthermore, EDX mapping (Fig. S3) confirms the homogeneous distribution of the constituent elements across the modified electrode surface.

XRD analysis (Fig. 1b) exhibits characteristic diffraction peaks at 2*θ* = 8.8°, 14.1°, 15.8°, and 17.7°, corresponding to the (100), (110), (101), and (200) crystal planes, respectively. These reflections indicate a lamellar MOF architecture with relatively ordered stacking, consistent with previously reported Co–BDC-based materials.^33^ Overall, these results confirm the successful formation of a crystalline MOF network.

Additionally, the ATR-FTIR spectrum confirms the coordination between cobalt ions and BDC linkers (Fig. 1c). The sharp peak observed at 3602 cm^−1^ is assigned to the O–H stretching vibration of hydroxyl groups. Within the carbonyl and carboxylate regions, the C=O stretching peak appears at 1654 cm^−1^, while the asymmetric and symmetric stretching vibrations of coordinated –COO^−^ groups are observed at 1572 cm^−1^ and 1372 cm^−1^, respectively. Furthermore, the peaks at 1497, 1099 and 748 cm^−1^ correspond to the C–H bending vibrations of the aromatic ring. Finally, the peak at 517 cm^−1^ is assigned to the Co–O stretching vibration, definitively confirming the Co–BDC framework formation.^34,35^

**Fig. 2:**
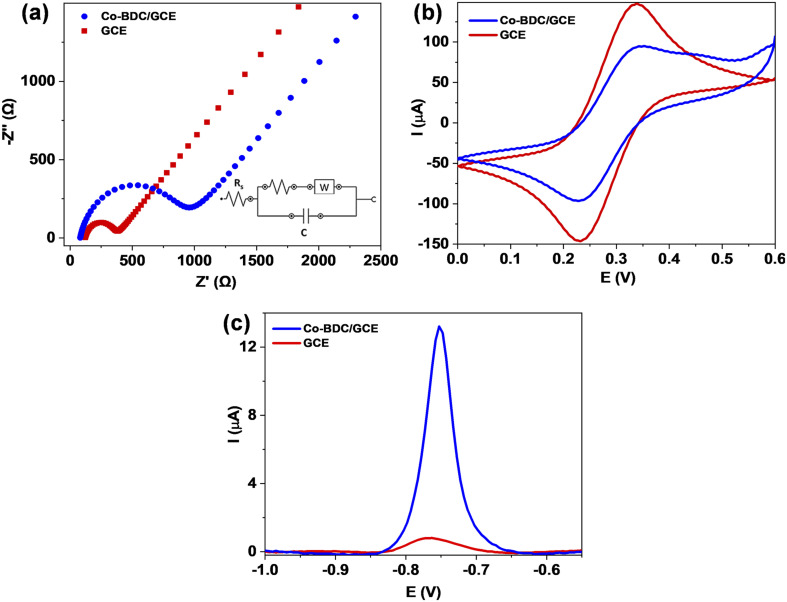
(a) EIS spectra; (b) cyclic voltammograms (CV) of the GCE and Co–BDC/GCE recorded in 5 mM [Fe(CN)_6_]^3−/4−^/0.1 M KCl at a scan rate of 0.1 V s^−1^; and (c) SWASV responses of the GCE and Co–BDC/GCE toward 20 ppb Cd^2+^ in 0.1 M KCl–HCl supporting electrolyte (pH 3.5) at an accumulation potential of −1.1 V for 120 s.

Electrochemical characterization of the electrodes was performed *via* electrochemical impedance spectroscopy (EIS) and cyclic voltammetry (CV) in a reversible K_3_[Fe(CN)_6_]/K_4_[Fe(CN)_6_] redox probe. As shown in Fig. 2a, the Nyquist plots of both the bare GCE and Co–BDC/GCE exhibit two distinct regions: a semicircle in the high-frequency range (corresponding to the charge transfer process) and a linear tail in the low-frequency region (indicative of a diffusion-controlled process). The calculated charge transfer resistance *R*_ct_ values were 242 Ω for the bare GCE and 845 Ω for the Co–BDC/GCE. This increase indicates that the presence of the Co–BDC layer slightly elevates the interfacial resistance, thereby hindering electron transfer at the electrode surface. These EIS findings are consistent with the CV results presented in Fig. 2b. Despite the higher charge transfer resistance of the Co–BDC/GCE, the well-defined redox peaks in its CV profile demonstrate that the reversible faradaic processes still proceed favorably. Consequently, this MOF remains highly suitable for electrochemical sensing applications, particularly owing to the excellent analyte adsorption capacity provided by its intrinsic porous architecture.

To further support the structural characteristics of the electrodeposited Co–BDC film, the electrochemically active surface area was estimated from the double-layer capacitance (*C*_dl_). The *C*_dl_ value was determined from the slope of the capacitive current difference (Δ*i* = (*i*_a_ − *i*_c_)/2) plotted against the scan rate, using cyclic voltammograms recorded in the non-faradaic potential region (0.05–0.20 V) in 0.1 M NaNO_3_ (Fig. S4). The ECSA was then calculated using the equation: ECSA = *C*_dl_/*C*_s_, where *C*_s_ is the specific capacitance of a smooth electrode surface, taken as 20 µF cm^−2^ according to the literature.^36,37^ The calculated ECSA of the Co–BDC/GCE reached 0.583 cm^2^, approximately 12.7 times higher than that of the bare GCE (0.046 cm^2^) (Table S1). This remarkable increase demonstrates that the electrodeposited Co–BDC film provides a substantially enlarged electrochemically accessible surface area compared with the bare electrode.

The surface porosity of the Co–BDC film was estimated from FESEM micrographs acquired at magnifications of 5000× and 20 000× using the ImageJ software for image analysis, following a previously reported image analysis procedure.^38,39^ The image processing procedure is illustrated in Fig. S5. Briefly, the FESEM images were converted into binary images using an appropriate threshold, and the projected pore area was quantified. The calculated projected surface porosity was 47.4% and 50.8% for the 5000× and 20 000× images, respectively, confirming the highly porous nature of the electrodeposited Co–BDC film. These results are consistent with the enlarged electrochemically accessible surface area obtained from the ECSA analysis.

The electrochemical signal of 20 ppb cadmium (*I*_p_), recorded by SWASV in 0.1 M KCl–HCl electrolyte (pH 3.5) at an accumulation potential of −1.1 V for 120 s on the modified electrode was approximately 15.3 times higher than that on the GCE (Fig. 2c), confirming the enhanced sensitivity of the Co–BDC MOF toward cadmium detection. The remarkable improvement can be attributed to the unique structural characteristics and adsorption capability of the Co–BDC framework.

The electrochemical sensing mechanism of Cd^2+^ at the Co–BDC-modified electrode is governed by a synergistic combination of mass transport, adsorption, coordination interactions, and redox reactions. In this study, the synthesized Co–BDC framework-comprising cobalt metal nodes coordinated with benzene-1,4-dicarboxylate ligands exhibits a hierarchical, coral-like morphology.

This unique architecture forms a three-dimensional open network that provides unobstructed ion-transport pathways, thereby accelerating the diffusion of Cd^2+^ ions from the bulk solution into the porous framework. Furthermore, compared to conventional particulate or sheet-like morphologies, this coral-like structure exposes a higher density of accessible active sites and an abundance of carboxylate functional groups, significantly enhancing the binding affinity of Cd^2+^ for the Co–BDC surface.

The direct growth of the Co–BDC film on the GCE also ensures strong interfacial contact between the active material and the conductive substrate, providing an effective electrical pathway during the electrochemical sensing process. In addition, the open branched network helps maintain accessible channels and minimizes aggregation of the active material, thereby improving its utilization efficiency. The carboxylate groups (–COO^−^) within the BDC ligands function as Lewis base sites that readily interact with Cd^2+^ ions *via* coordination bonds or electrostatic attraction.^40,41^ Consequently, the synergistic combination of the hierarchical coral-like morphology, the porous framework, and the abundance of carboxylate adsorption sites significantly accelerates mass transfer and enhances ion accumulation, thereby boosting the electrochemical sensing performance of the Co–BDC modified electrode for Cd^2+^ detection.

To experimentally validate the proposed sensing mechanism, additional SWV and ATR-FTIR experiments were performed. As shown in Fig. S6a, no Cd reduction peak was observed when the Co–BDC/GCE was directly measured in a 500 ppb Cd^2+^ solution without a prior adsorption step. In contrast, a distinct reduction peak appeared after immersing the electrode in the Cd^2+^ solution at open-circuit potential for 300 s, confirming the preconcentration of Cd^2+^ on the Co–BDC surface before electrochemical reduction. A characteristic Cd signal was observed in the EDX spectrum after Cd^2+^ adsorption (Fig. S6b), confirming the presence of Cd species on the Co–BDC surface. Furthermore, ATR-FTIR spectra recorded before and after Cd^2+^ adsorption (Fig. S6c) provided direct evidence of the chemical interactions between the analyte and the MOF framework. Following Cd^2+^ exposure, the asymmetric carboxylate stretching vibration (*ν*_as_) shifted from 1572 cm^−1^ to 1575 cm^−1^, while the symmetric stretching vibration (*ν*_s_) shifted from 1372 cm^−1^ to 1387 cm^−1^, resulting in a decrease in the band separation (Δ*ν* = *ν*_as_ − *ν*_s_) from 200 to 188 cm^−1^. This reduction in Δ*ν* indicates a modification in the coordination mode of the carboxylate groups, potentially shifting toward a more symmetric or chelating bidentate configuration upon binding with Cd^2+^. Concurrently, the disappearance of the peak at 3602 cm^−1^ (ascribed to O–H stretching of hydroxyl groups), together with perturbations in the aromatic ring vibrations and out-of-plane C–H deformations (around 1497 cm^−1^), demonstrate the active participation of oxygen-containing functional groups in coordinating the captured Cd^2+^ ions.^42^ Overall, these results strongly support the proposed adsorption-assisted sensing mechanism.

The electrochemical behavior of Cd^2+^ at the Co–BDC/GCE was further investigated by cyclic voltammetry at scan rates ranging from 0.01 V s^−1^ to 0.10 V s^−1^ in 0.1 M KCl–HCl solution (pH 3.5) containing 2 ppm Cd^2+^. As shown in Fig. S7, the peak current exhibited a linear relationship with both scan rate (*ν*) and *ν*^1/2^, following the equations: *I*_pc_ = 879.549*ν* + 12.469 (*R*^2^ = 0.9862) and *I*_pc_ = 367.022*ν*^1/2^ − 20.719 (*R*^2^ = 0.9717), respectively. These results indicate that the electrochemical response is strongly dependent on the scan rate and involves both mass transport and surface-related processes. Furthermore, the plot of log *I*_p_*versus* log *ν* yielded a slope of 0.697, which lies between the theoretical values of 0.5 and 1.0 corresponding to diffusion-controlled and adsorption-controlled processes, respectively. This indicates that the sensing process is governed by a mixed diffusion–adsorption mechanism.^43,44^ The porous interconnected Co–BDC structure promotes the diffusion of Cd^2+^ ions through the framework, while the abundant carboxylate groups provide adsorption and coordination sites for Cd^2+^ accumulation. Consequently, the enhanced electrochemical response arises from the synergistic contribution of diffusion-assisted mass transport and surface accumulation of Cd^2+^ within the porous Co–BDC network. The electron-transfer behavior of Cd^2+^ at the Co–BDC/GCE was further examined by plotting *E*_p_ against log *ν* (Fig. S7e). A linear relationship was obtained (*E*_pc_ = −0.069 log *ν* − 1.066, *R*^2^ = 0.9889), indicating a quasi-reversible electrode process. According to Laviron's equation, the slope corresponds to a two-electron transfer process (*n* = 2) for the Cd^2+^/Cd redox reaction, consistent with the electrochemical deposition and stripping mechanism of Cd^2+^ on the electrode surface.

In addition, the peak-to-peak separation (Δ*E*_p_) was analyzed to evaluate the reversibility of the Cd^2+^/Cd redox process (Fig. S7). The Δ*E*_p_ value increased from 205.9 mV at 0.01 V s^−1^ to 269.2 mV at 0.10 V s^−1^, significantly exceeding the theoretical value for a reversible two-electron process (Δ*E*_p_ ≈ 29.5 mV) and indicating a scan-rate-dependent quasi-reversible behavior. The increase in Δ*E*_p_ mainly originated from the cathodic branch, where *E*_pc_ shifted toward more negative potentials with increasing scan rate, whereas *E*_pa_ remained nearly unchanged. This suggests that the kinetic limitation is primarily associated with the Cd^2+^ reduction/deposition step, which involves electron transfer coupled with the nucleation and growth of metallic Cd on the Co–BDC surface. Therefore, the scan-rate-dependent increase in Δ*E*_p_ confirms the quasi-reversible nature of the Cd^2+^/Cd redox process on the Co–BDC electrode.

### 3.2. Influence of MOF synthesis conditions

**Fig. 3:**
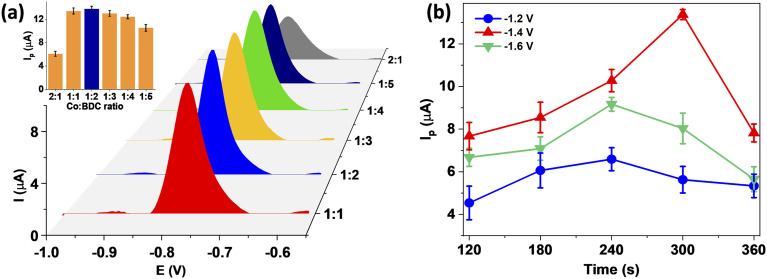
(a) SWASV responses of Co–BDC/GCEs fabricated with different Co^2+^ : BDC precursor ratios in 0.1 M KCl–HCl solution (pH 3.5) containing 20 ppb Cd^2+^; and (b) Cd^2+^ peak heights of Co–BDC/GCE prepared at different deposition potentials (−1.2 V, −1.4 V, and −1.6 V) for deposition times ranging from 120 s to 360 s.

Variations in synthesis parameters-including the precursor concentration ratio (denoted as Co^2+^ : BDC), deposition potential, and deposition time – significantly influence the characteristics of the Co–BDC layer formed on the GCE surface and, consequently, the resulting Cd^2+^ detection signal. A noticeable decrease in the current response toward 20 ppb Cd^2+^ is observed when an excess of Co^2+^ is used relative to the BDC ligand (Co^2+^ : BDC = 2 : 1). This reduction is likely attributed to the preferential formation of Co(OH)_2_ rather than the growth of the Co–BDC MOF under cathodic polarization, which diminishes the electroactive properties of the electrode surface. In contrast, higher responses are obtained at Co^2+^ : BDC ratios of 1 : 1 and 1 : 2, whereas the signal slightly decreases again when the Co^2+^ : BDC ratio is further reduced to 1 : 5 (Fig. 3a). When the BDC concentration is substantially higher than that of Co^2+^, incomplete nucleation or insufficient metal–ligand coordination may occur, resulting in a thinner or poorly interconnected MOF film with diminished electrochemical activity. Therefore, a Co^2+^ : BDC ratio of 1 : 2 was selected as the optimal condition for subsequent experiments.

**Fig. 4:**
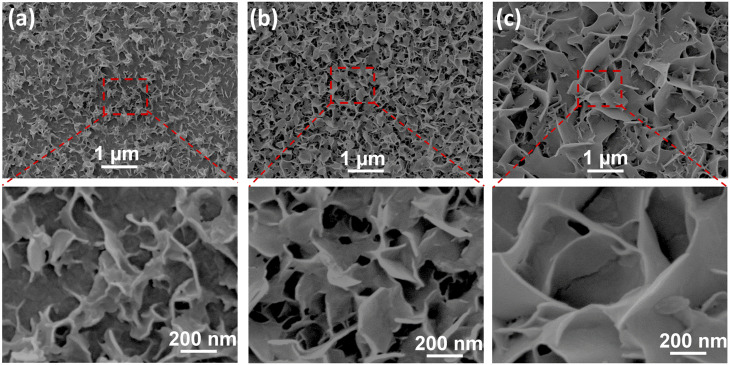
FESEM images of Co–BDC/GCE electrodes synthesized at deposition times of (a) 120 s, (b) 300 s, and (c) 360 s under a deposition potential of −1.4 V.

The SWASV curves and the dependence of the Cd^2+^ signal on the applied potential and deposition time during Co–BDC/GCE synthesis are presented in Fig. S8 and 3b. Among the three tested deposition potentials (−1.2 V, −1.4 V, −1.6 V), the electrodes prepared at −1.4 V exhibited the highest cadmium stripping signals across all deposition times (120–360 s). At −1.2 V, as shown in the SEM image (Fig. S9a), the resulting surface exhibits large, irregular crystalline domains with a poorly defined morphology. This phenomenon can be attributed to the predominant formation of Co(OH)_2_ rather than the Co–BDC MOF, which stems from the insufficient release of OH^−^ ions – levels that are inadequate to deprotonate H_2_BDC and promote the development of the MOF framework. Conversely, at a more negative potential of −1.6 V (Fig. S9b), the reaction kinetics proceed too rapidly, leading to uncontrolled Co–BDC crystal growth and the concomitant formation of Co(OH)_2_. As a result, large plate-like structures are formed instead of the uniform porous film observed at −1.4 V (Fig. S8b). This irregular morphology substantially decreases the density of active sites available for ion adsorption and electron transfer, thereby lowering the Cd^2+^ response compared to the electrodes prepared at −1.4 V. Therefore, −1.4 V was selected as the optimal deposition potential. At −1.4 V, the maximum Cd^2+^ peak current was achieved at a deposition time of 300 s, corresponding to the formation of a highly porous Co–BDC nanosheet network that maximizes Cd^2+^ accumulation (Fig. 4b). Shorter (120 s) and longer (360 s) deposition times yielded lower signals due to incomplete surface coverage Fig. 4a and reduced porosity from nanosheet thickening Fig. 4c, respectively. Thus, 300 s was chosen as the optimal deposition time.

### 3.3. Optimization of detection conditions

**Fig. 5:**
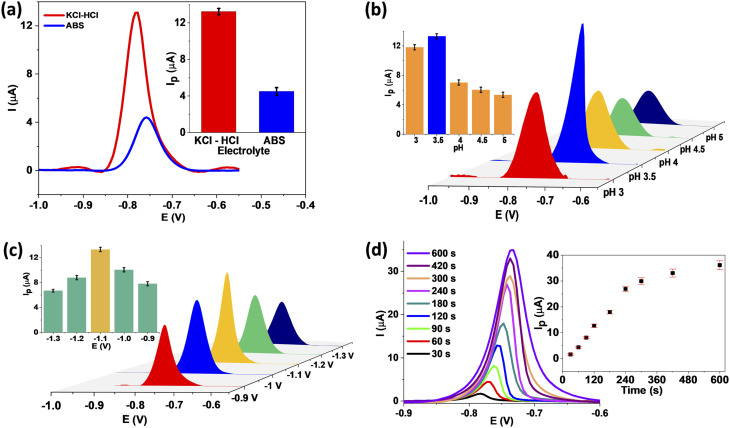
SWASV responses of 20 ppb Cd^2+^: (a) in ABS and KCl–HCl solutions at pH 3.5, (b) in KCl–HCl at different pH values, (c) at various deposition potentials, and (d) effect of accumulation time on the peak current.

KCl–HCl and acetate buffer solution (ABS) are two of the most widely used supporting electrolytes for the electrochemical detection of heavy metal ions. In this study, the stripping signal of 20 ppb Cd^2+^ was clearly observed in both electrolytes at pH 3.5. However, the peak current obtained in the KCl–HCl solution was approximately three times higher than that in ABS (Fig. 5a), indicating more favorable ionic conductivity and faster electron-transfer kinetics. Therefore, when utilizing the Co–BDC/GCE as the working electrode, the KCl–HCl system was superior for Cd^2+^ detection, the KCl–HCl system was selected as the optimal supporting electrolyte for use with the Co–BDC/GCE.

The pH of the supporting electrolyte significantly influences the Cd^2+^ stripping peak current. As shown in Fig. 5b, the maximum peak current was achieved at pH 3.5 of KCl–HCl electrolyte. At a lower pH of 3.0, the peak current decreases slightly, which can be attributed to the competitive adsorption between excess H^+^ and Cd^2+^ ions for the active sites on the electrode surface, as well as interference from the hydrogen evolution reaction (HER) that hinders cadmium accumulation. In less acidic media (pH ≥ 4), the Cd^2+^ response dropped markedly. This reduction is likely because cadmium ions begin to undergo hydrolysis or form complex species at higher pH values, which are unfavorable for interaction with the MOF-modified surface, thereby diminishing the analytical signal. Consequently, pH 3.5 was selected for further investigations.

The preconcentration potential and accumulation time for Cd^2+^ detection were optimized using 20 ppb Cd^2+^ at pH 3.5 in 0.1 M KCl–HCl. As illustrated in Fig. 5c, when the deposition potential was shifted from −0.9 V to −1.3 V, the peak current progressively increased, reaching its maximum value at −1.1 V. When the potential became more negative than −1.1 V, the stripping signal decreased notably. This initial trend is ascribed to the increased overpotential (energy input) that drives efficient cadmium preconcentration. However, when the applied potential becomes excessively negative (−1.2 V to −1.3 V), the concomitant hydrogen evolution reaction generates bubbles that partially block the electrode surface, thereby limiting cadmium electrodeposition and severely reducing the subsequent SWASV signal. Furthermore, the effect of accumulation time (*t*_dep_) on the 20 ppb Cd^2+^ response is demonstrated in Fig. 5d. As *t*_dep_ extended from 30 seconds to 300 seconds, the peak current increased significantly. However, the rate of current increase decelerated as the time approached 300 s, presumably due to the surface saturation of the electrodeposited cadmium on the electrode matrix. To balance analysis time and maximize detection sensitivity, an accumulation time of 300 s was selected for the subsequent analytical studies.

### 3.4. Calibration curve and limit of detection for Cd^2+^ detection

**Fig. 6:**
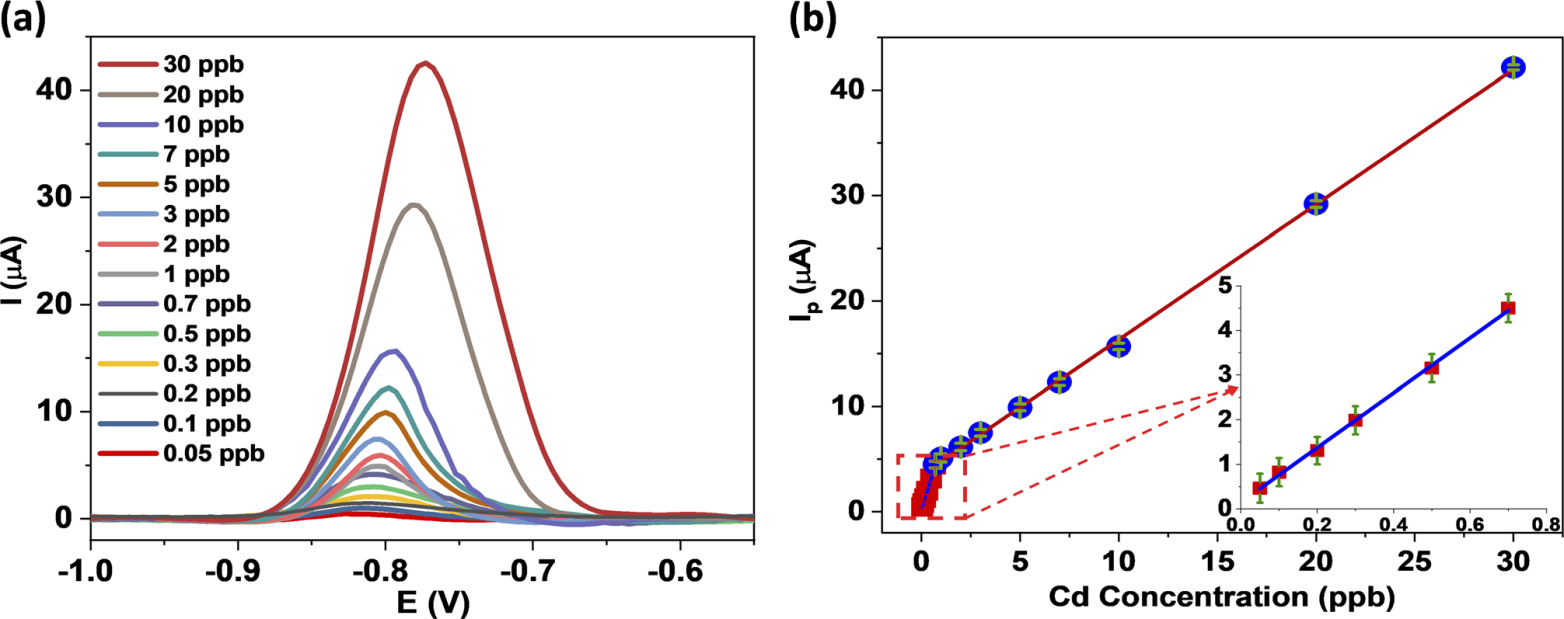
(a) SWASV responses of the Co–BDC/GCE in Cd^2+^ solutions with concentrations ranging from 0.05 ppb to 30 ppb and (b) the corresponding calibration curve of Cd^2+^ peak height *versus* concentration. All measurements were performed in 0.1 M KCl–HCl solution (pH 3.5) at a deposition potential of −1.1 V for 300 s.

The SWASV responses at various concentrations of Cd^2+^ ranging from 0.05 ppb to 30 ppb recorded on the developed electrode are presented in Fig. 6.

The stripping peak currents increased proportionally with the Cd^2+^ concentration across two distinct linear dynamic ranges: 0.05–0.7 ppb and 0.7–30 ppb. The corresponding linear regression equations are: *I* (µA) = 6.143*C* (ppb) + 0.146 (*R*^2^ = 0.9987) and *I* (µA) = 1.282*C* (ppb) + 3.507 (*R*^2^ = 0.9995), respectively.

The limit of detection (LOD) and limit of quantification (LOQ) for Cd^2+^ using the Co–BDC/GCE electrode, were calculated from the calibration curve in the lower concentration range (0.05–0.7 ppb), according to the equations: LOD = 3.3*σ*/*b* and LOQ = 10*σ*/*b*, where *σ* is the standard deviation of the residuals and *b* is the slope of the calibration plot. The calculated LOD and LOQ values were 0.035 ppb and 0.105 ppb, respectively. The obtained LOD is lower than or comparable to those of previously reported Cd^2+^ sensors (Table 1). Notably, the proposed Co–BDC/GCE features a simple composition and can be rapidly fabricated *via* direct electrodeposition. This binder-free approach yields a highly uniform and porous film while eliminating insulating binders that typically hinder interfacial electron transfer.

**Table 1:** Comparison of Cd^2+^ detection performance with previous studies

Electrode	Fabrication method	Method	Linear range (ppb)	LOD (ppb)	Sensitivity (µA ppb^−1^)	Stability	Real-sample performance	Ref.
CUiO-66/Bi/GCE	Drop-casting and electrochemical	SWASV	10–50	1.66	0.07935	—, 28 days	Recoveries ranging from 91% to 105%	3
SPAN@UiO-66 NH_2_/SPCE	Drop-casting	SWASV	0.5–100	0.17	0.3536	RSD = 4.1%, 30 days (95%)	Recoveries ranging from 96.6% to 108.1%	4
MIL-88B(Fe)-NH_2_/GCE	Drop-casting	DPV	0.281–112.4	0.0225	0.004	—	Recoveries ranging from 97.2% to 104.0%	5
NH_2_-MIL-88(Fe)-rGO/GCE	Drop-casting	DPASV	0.562–5.62	0.551	1.69	RSD = 3.78%, 15 days (94.1%)	Recoveries ranging from 98.1% to 100.2%	6
l-Au–MOFs–GO/GCE	Drop-casting	DPV	80–560	20.8	0.004	RSD ≈ 2%	Recoveries ranging from 80% to 100%	7
ZIF-67/rGO/Grafoil	Drop-casting	SWASV	5–100	2.93	0.485	RSD ≈ 2%	—	8
MIL-53(Fe)/GCE	Drop-casting	DPV	16.9–50.6	1.8	0.0151	—	—	45
UiO-66-NH_2_/MWCNTs/GCE	Drop-casting	DPSV	0.5–170	0.2	0.1103	RSD = 4.6%, 14 days (91.7%)	Recoveries ranging from 95.1% to 107.5%	46
Bi-NPs/NC/GP	Drop-casting	SWASV	0.5–1200	0.05	0.02928	RSD < 1%, 30 days (91.9%)	Recoveries ranging from 105% to 105.8%	47
**Co–BDC/GCE**	**Electrochemical synthesis**	**SWASV**	**0.05–30**	**0.035**	**6.143**	**RSD = 1.35%, 50 days (65%)**	**Recoveries ranging from 93.1% to 110.1%**	**This work**

### 3.5. Reproducibility, selectivity and stability of Co–BDC/GCE

**Fig. 7:**
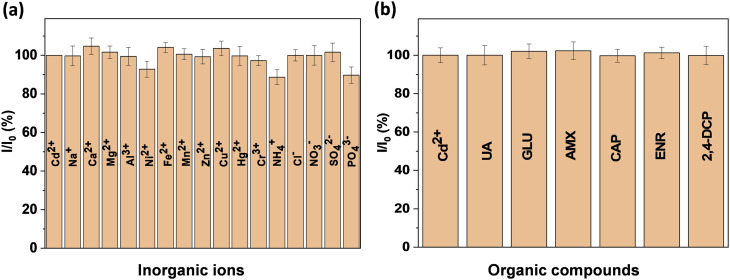
Effect of (a) inorganic and (b) organic interfering species on the Cd^2+^ peak height (20 ppb). All measurements were performed in 0.1 M KCl–HCl (pH 3.5) at a deposition potential of −1.1 V for 300 s.

To evaluate the sensor-to-sensor reproducibility, eight independently prepared Co–BDC/GCEs were used to measure the signal of 20 ppb Cd^2+^ under identical optimized conditions (Fig. S10). The relative standard deviation (RSD) of the peak currents was calculated to be 1.35%, confirming the excellent reproducibility of the fabrication process and the high reliability of the developed analytical method. The interference of common inorganic ions and several organic compounds on the Cd^2+^ signal was investigated to assess the selectivity of the electrode. Fig. 7 illustrates the percentage of the Cd^2+^ signal retained in the presence of various substances – including Na^+^, Ca^2+^, Mg^2+^, Al^3+^, Ni^2+^, Fe^2+^, Mn^2+^, Zn^2+^, Cu^2+^, Hg^2+^, Cr^3+^, NH_4_^+^, Cl^−^, NO_3_^−^, SO_4_^2−^, PO_4_^3−^, uric acid (UA), glucose (GLU), amoxicillin (AMX), chloramphenicol (CAP), enrofloxacin (ENR), and phenol compound (2,4-DCP) each at 100-fold the Cd^2+^ concentration. None of these substances caused a significant deviation in the Cd^2+^ response, demonstrating the robust anti-interference capability of the Co–BDC/GCE. This exceptional selectivity toward Cd^2+^ is primarily attributed to its specific ionic radius and distinct coordination thermodynamics, which facilitate preferential binding to the Co–BDC surface. Consequently, the developed sensor holds great promise for reliable Cd^2+^ detection in complex real-world matrices.

The stability of the fabricated electrode was assessed by recording the Cd^2+^ response over an extended period while the electrodes were stored in a desiccator at room temperature to prevent moisture exposure (Fig. S11). The response gradually decreased to approximately 82%, 75%, 69%, and 65% of the initial value after 20, 30, 40, and 50 days, respectively. Although a gradual decline in response was observed during prolonged storage, the electrode maintains acceptable long-term stability for practical applications. More importantly, its outstanding sensitivity and the simple one-step electrodeposition process allow for rapid regeneration of a fresh Co–BDC/GCE within minutes, effectively reducing the reliance on extended storage stability in real-world use.

### 3.6. Real sample analysis

The practical applicability of the prepared electrode was further demonstrated by determining Cd^2+^ in real samples, including lake water, river water, and vegetable samples (Fig. S12 and Table 2). Cd^2+^ concentrations of 0.12 ppb and 0.05 ppb were detected by the proposed Co–BDC/GCE sensor in West Lake and the Cau River water, respectively. Notably, both concentrations were below the LOD of the reference ICP-MS method (0.16 ppb), highlighting the superior sensitivity of the proposed Co–BDC/GCE (LOD 0.035 ppb) for trace Cd^2+^ determination, which is consistent with the excellent preconcentration capability of the porous MOF. To further evaluate its analytical performance in real sample matrices, spike recovery experiments were performed using the collected samples. As summarized in Table 2, satisfactory recoveries ranging from 93.1% to 110.1% were obtained, with relative errors below 2.5% compared with the ICP-MS method, demonstrating good accuracy and negligible matrix interference. Furthermore, the Cd^2+^ concentration determined in the vegetable sample by the proposed method was in good agreement with that obtained by the validated ICP-MS method. Crucially, the Cd^2+^ concentrations in all tested samples were well below the permissible limits set by the World Health Organization (WHO) and the European Union (EU). These results clearly demonstrate the reliability and practical applicability of the proposed sensor for Cd^2+^ determination in real samples, highlighting its great potential for environmental monitoring and food analysis.

**Table 2:** Determination of Cd^2+^ in real samples using the Co–BDC/GCE sensor and comparison with ICP-MS results (*C*_Found_: Cd^2+^ concentration measured by SWASV; *C*_Sample_: Cd^2+^ concentration in the original sample)

Real sample	*C*_Spike_ (ppb)	*C*_Found_		*C*_Net_ = *C*_Found_ − *C*_sample_	Recovery (%) = 100 × *C*_Net_/*C*_Spike_	Relative error (%) = 100 × ∣*C*_SWASV_ − *C*_ICP-MS_∣/*C*_ICP-MS_	(WHO/EU) permissible limit
		SWASV (Co–BDC/GCE)	ICP-MS				
**West lake (Hanoi)**	0	0.120 ppb	<LOD (0.16 ppb)	—	—		3 ppb, Guidelines for drinking-water quality: fourth edition incorporating the first and second addenda (WHO)
Add 0.5 ppb	0.5	0.588 ppb	0.6 ppb	0.468 ppb	93.6	2%	
Add 1 ppb	1	1.051 ppb	1.03 ppb	0.931 ppb	93.1	2.04%	
**Cau river (Thai Nguyen)**	0	0.05 ppb	<LOD (0.16 ppb)	—	—		
Add 0.5 ppb	0.5	0.531 ppb	0.52 ppb	0.481 ppb	96.2	2.12%	
Add 1 ppb	1	1.151 ppb	1.18 ppb	1.101 ppb	110.1	2.46%	
**Sweet potato vegetable (Thai Nguyen)**	0	0.385 ppb	—	—	—		30 µg kg^−1^, Commission Regulation (EU) 2021/1323 (European Union)
		21.37 µg kg^−1^	21.54 µg kg^−1^				
Add 0.5 ppb	0.5	0.911 ppb	0.895 ppb	0.526 ppb	105.2	1.79%	

## 4. Conclusion

A porous coral-like Co–BDC MOF nanostructure was successfully synthesized on the GCE surface *via* a simple and rapid one-step cathodic chronoamperometry method and applied to Cd^2+^ detection. The surface morphology of the modified electrode, and consequently the Cd^2+^ response signal, strongly depended on the fabrication conditions, including the applied potential, deposition time, and metal-to-linker ratio. Under the optimized analytical condition, the fabricated Co–BDC/GCE exhibited a Cd^2+^ signal that was 15.3-fold higher than that of the bare GCE, together with excellent reproducibility, long-term stability, a low detection limit, and high selectivity. Furthermore, the sensor demonstrated reliable performance in real samples, including surface water and vegetable samples. The results highlight the strong potential of the proposed Co–BDC-based electrochemical sensor for practical monitoring of Cd^2+^ in environmental and food samples.

## Author contributions

Dinh Dung Luong: data curation, formal analysis, investigation, writing – original draft. Thi Hai Yen Pham: formal analysis, investigation, methodology, writing – original draft. Tien Dat Doan: data curation, investigation. Nhung Hac Thi: formal analysis, investigation. Ho Thi Oanh: investigation, validation. Hong Tham Nguyen: investigation. Thi Thu Ha Vu: conceptualization, investigation. Tuyen Van Nguyen: conceptualization, validation. Bui Dinh Tu: methodology, validation. Thi Kim Dung Hoang: project administration, visualization. Mai Ha Hoang: methodology, project administration, resources, supervision, writing – review and editing.

## Conflicts of interest

The authors declare that they have no known competing financial interests or personal relationships that could have appeared to influence the work reported in this paper.

## Supplementary Material

RA-OLF-D6RA02923J-s001

## Data Availability

The data supporting this article have been included as part of the supplementary information (SI). Supplementary information is available. See DOI: https://doi.org/10.1039/d6ra02923j.
