# Supplementary material for: Coral-like 3D porous Co–BDC nanostructures synthesized by simple one-step chronoamperometry for electrochemical detection of Cd2+
Source: RSC Adv. 2026 Jul 20;16(38):41802–13. doi: 10.1039/d6ra02923j (PMC13384233; doi:10.1039/d6ra02923j)
Supplement: Supplementary file 1 [file RA-016-D6RA02923J-s001.pdf]

Supplementary Information (SI) for RSC Advances.
This journal is © The Royal Society of Chemistry 2026

Coral-Like 3D Porous Co-BDC Nanostructures Synthesized by Simple One-Step Chronoamperometry for Electrochemical Detection of Cd^{2+}

Dinh Dung Luong,[ab] Thi Hai Yen Pham,[a] Tien Dat Doan,[a] Nhung Hac Thi,[a] Ho Thi Oanh,[a] Hong Tham Nguyen,[a] Thi Thu Ha Vu,[a] Tuyen Van Nguyen,[a] Bui Dinh Tu,[b] Thi Kim Dung Hoang,[c] Mai Ha Hoang *[a]

[a] *Institute of Chemistry, Vietnam Academy of Science and Technology (VAST), 18 Hoang Quoc Viet, Nghia Do Ward, Hanoi, 10000, Vietnam*

[b] *Faculty of Engineering Physics and Nanotechnology, VNU University of Engineering and Technology, Vietnam National University, 144 Xuan Thuy, Cau Giay, Hanoi, 10000, Vietnam*

[c] *Institute of Advanced Technology, Vietnam Academy of Science and Technology, No. 1B, TL29 Str., An Phu Dong Ward, Ho Chi Minh City, 70000, Vietnam*

Email: Hoangmaiha@ich.vast.vn

Supporting Information

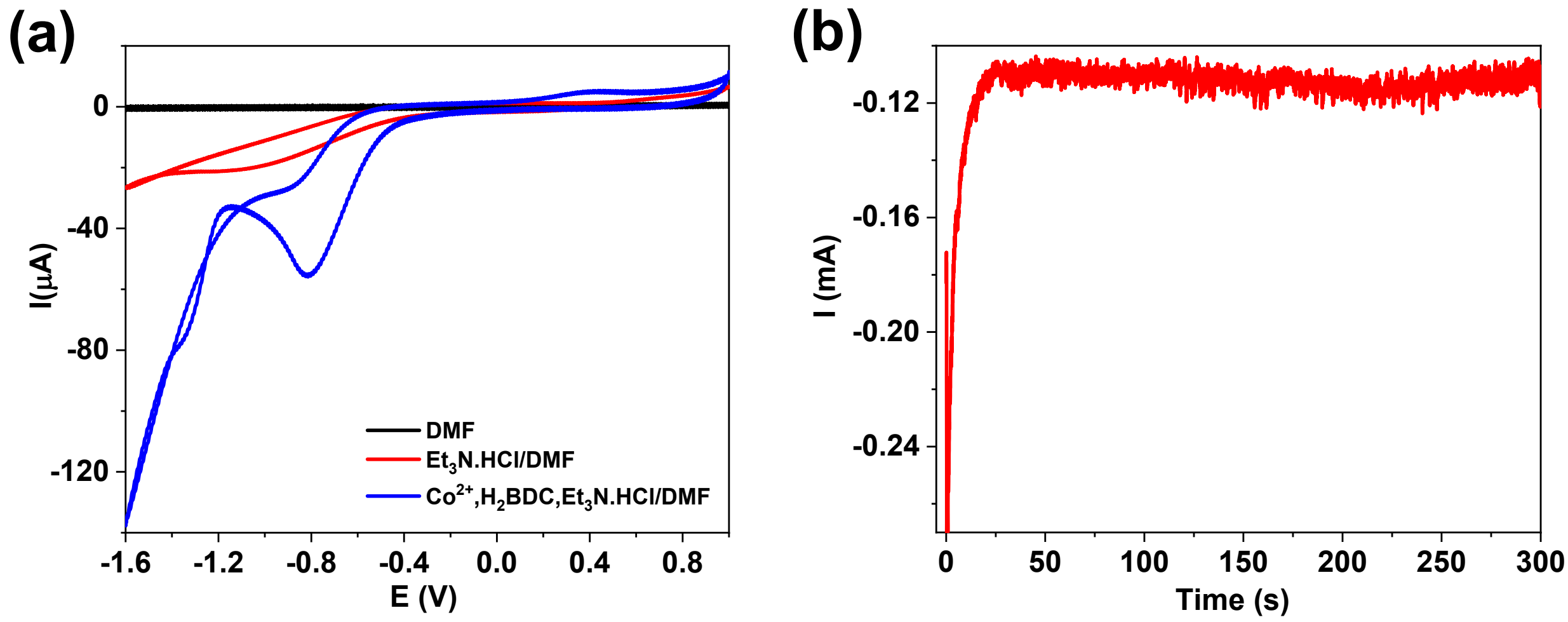

Fig. S1 (a) Cyclic voltammograms of the GCE recorded in the precursor solutions within the potential range of 1.0 to -1.6 V at a scan rate of 0.1 V s^{-1}; (b) Chronoamperometric curve for the electrodeposition of Co-BDC on the GCE at -1.4 V for 300 s.

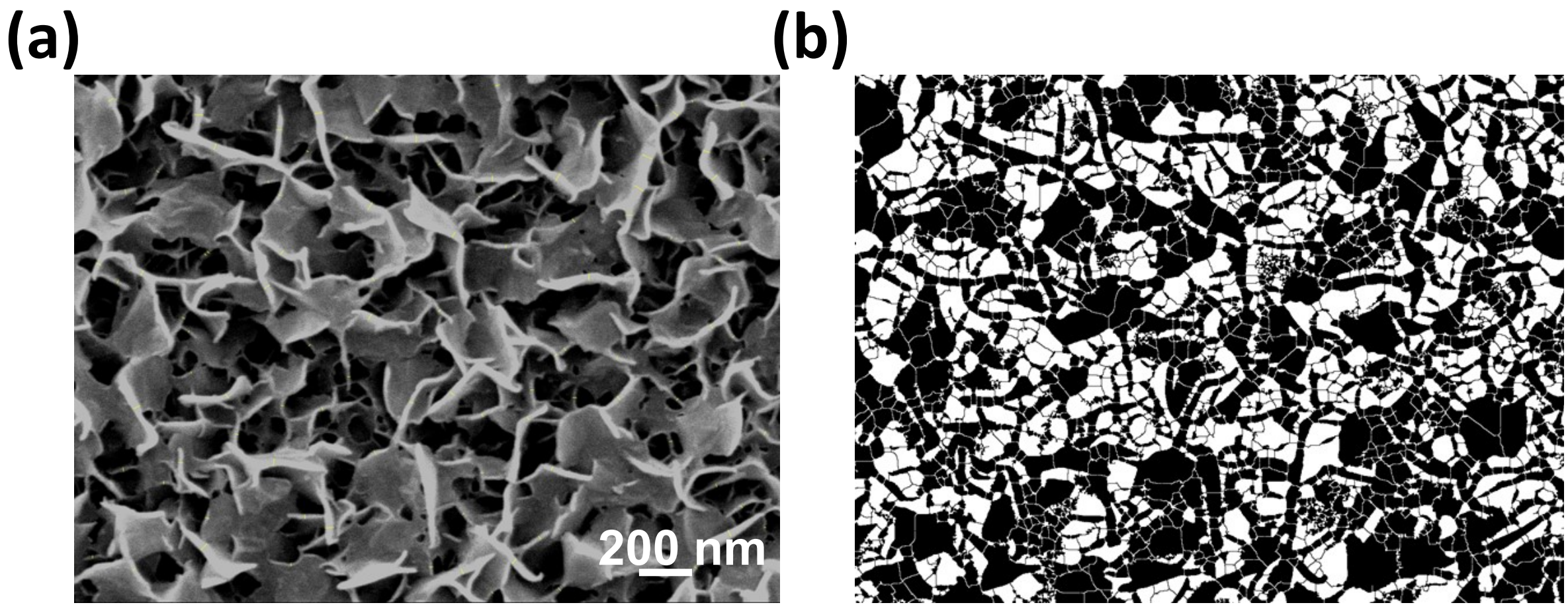

Fig. S2 Fiji-based image analysis of the electrodeposited Co-BDC film. (a) FESEM image (50.000×) showing straight-line measurements of the apparent nanosheet thickness. (b) Thresholded binary image used for pore size and surface porosity analysis.

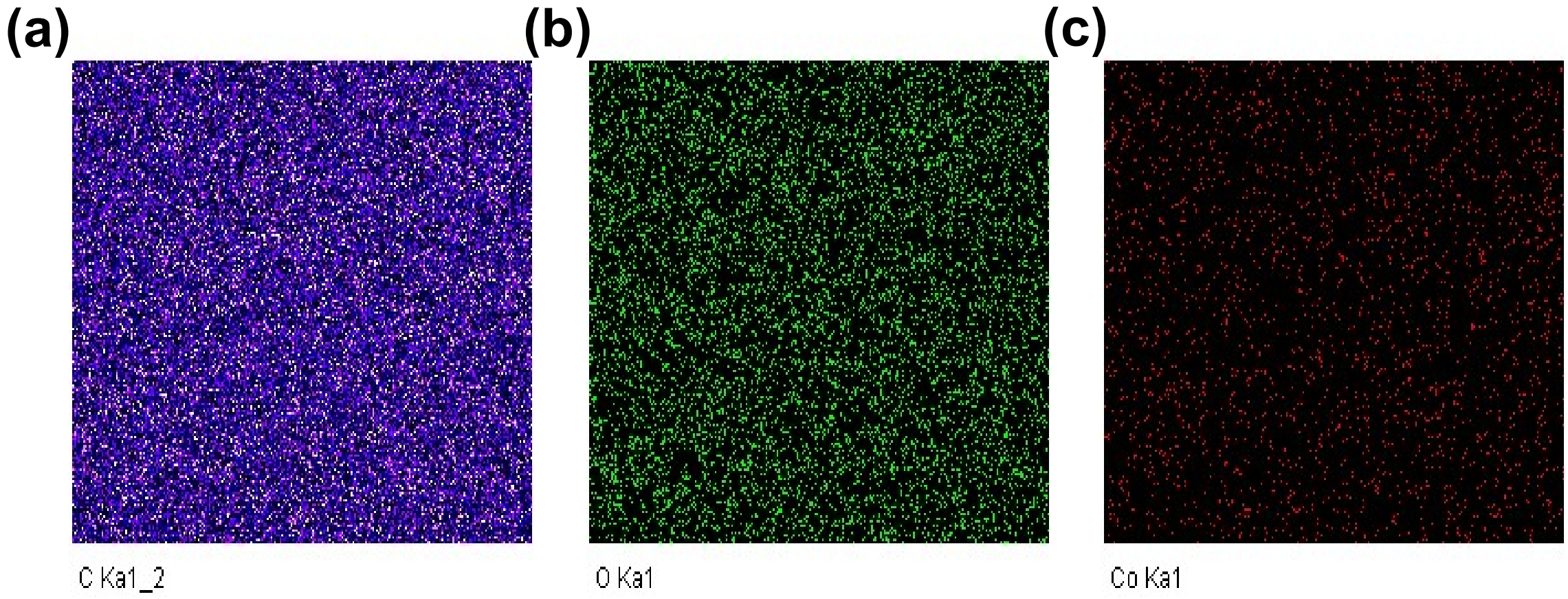

Fig. S3 FESEM-EDS mapping of the Co-BDC/GCE surface showing the distribution of (a) C, (b) O, and (c) Co elements.

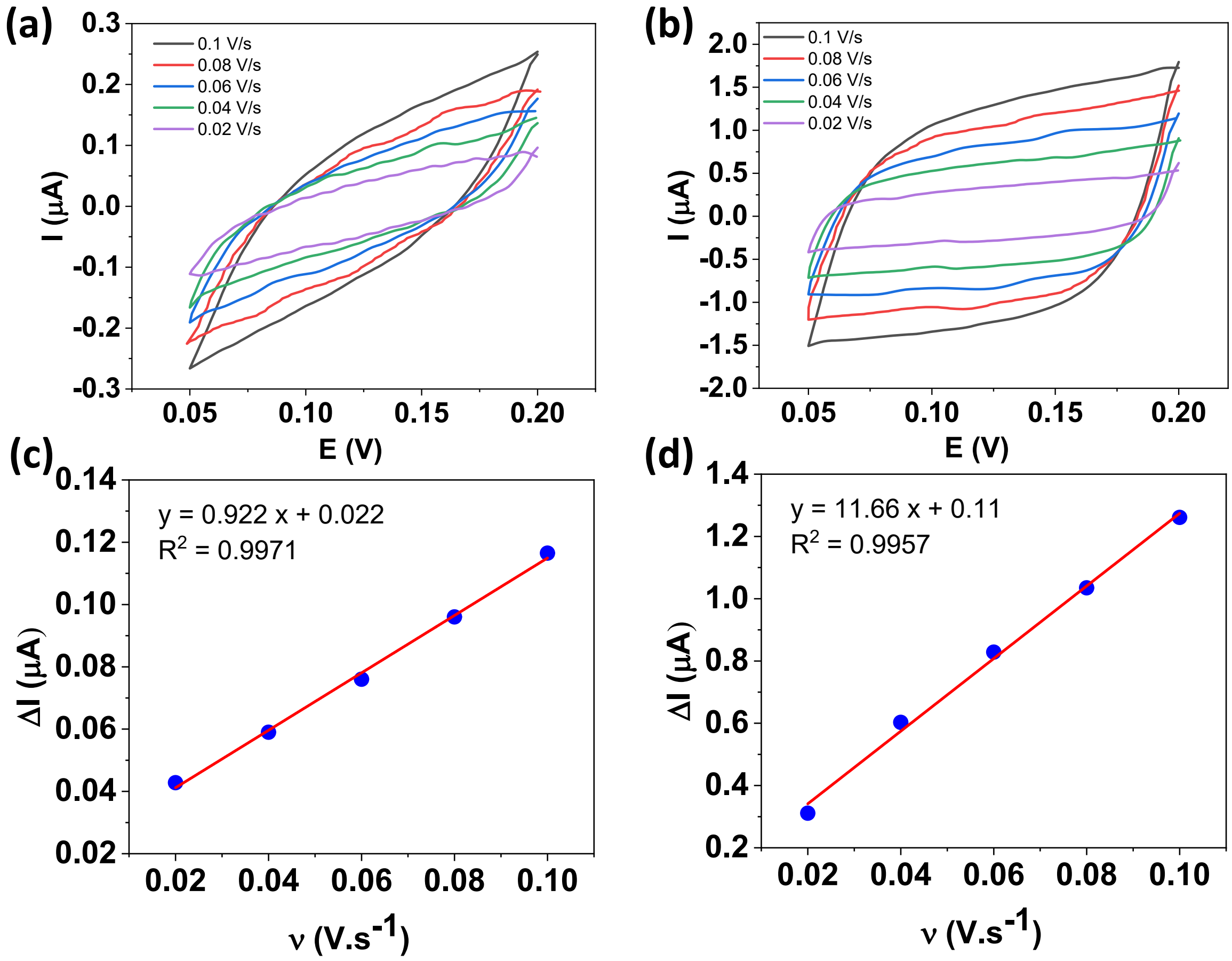

Fig. S4 Cyclic voltammograms of (a) bare GCE and (b) Co-BDC/GCE recorded in 0.1 M $NaNO_3$ over the potential range of 0.05-0.20 V at different scan rates. (c, d) Plots of the capacitive current difference ($\Delta i = (i_a - i_c)/2$) versus scan rate for the bare GCE (c) and Co-BDC/GCE (d), respectively.

Table S1. Electrochemical parameters used for ECSA determination of the bare GCE and Co-BDC/GCE electrodes.

Electrode	C_{dl} (µF)	ECSA (cm^2)
GCE	0.922	0.046
Co-BDC/GCE	11.660	0.583

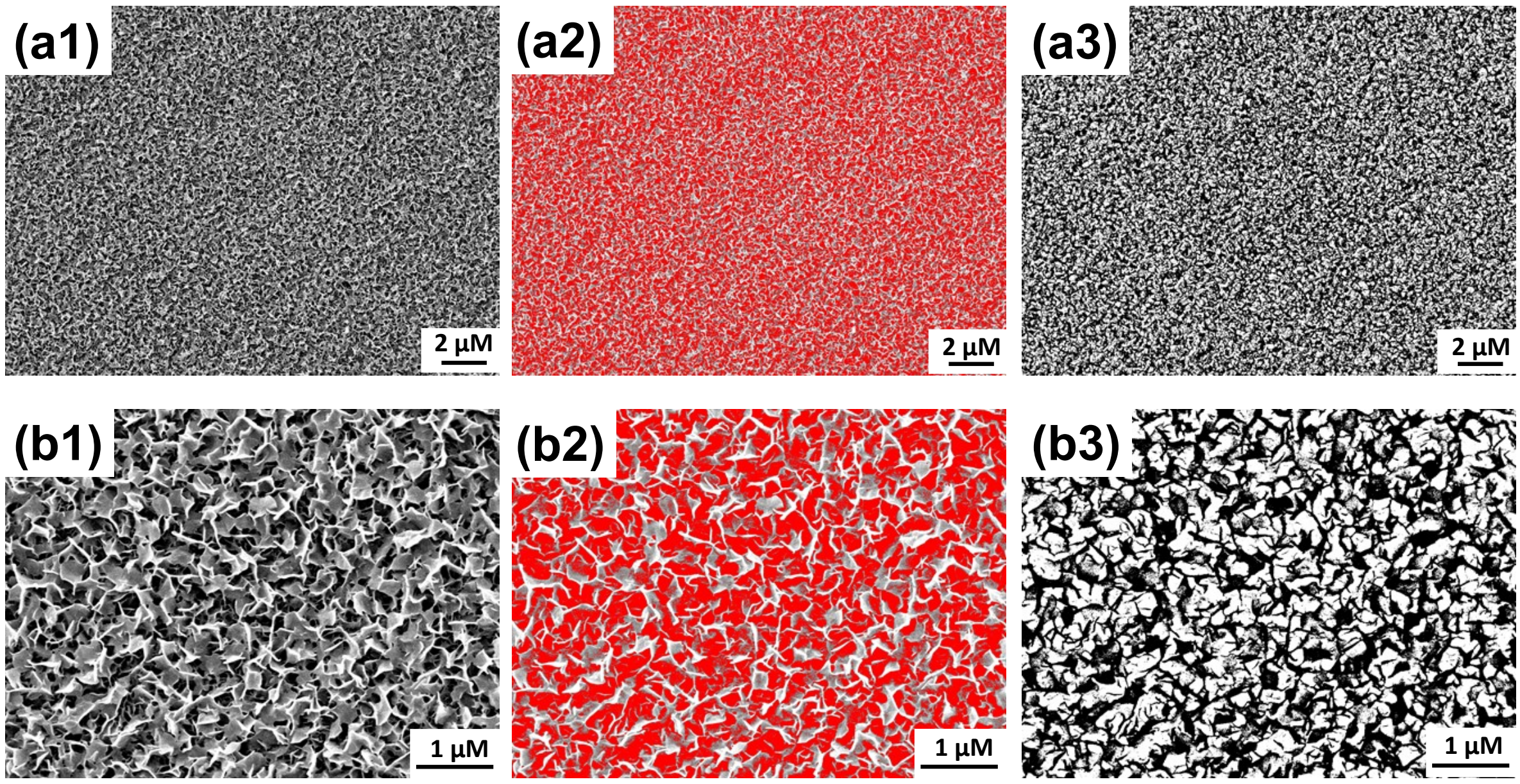

Fig. S5 Image processing procedure for surface porosity analysis of the Co-BDC film using ImageJ. FESEM micrographs acquired at (a) 5.000× and (b) 20.000× magnification; (a1, b1) original FESEM images; (a2, b2) thresholded images; and (a3, b3) processed binary images

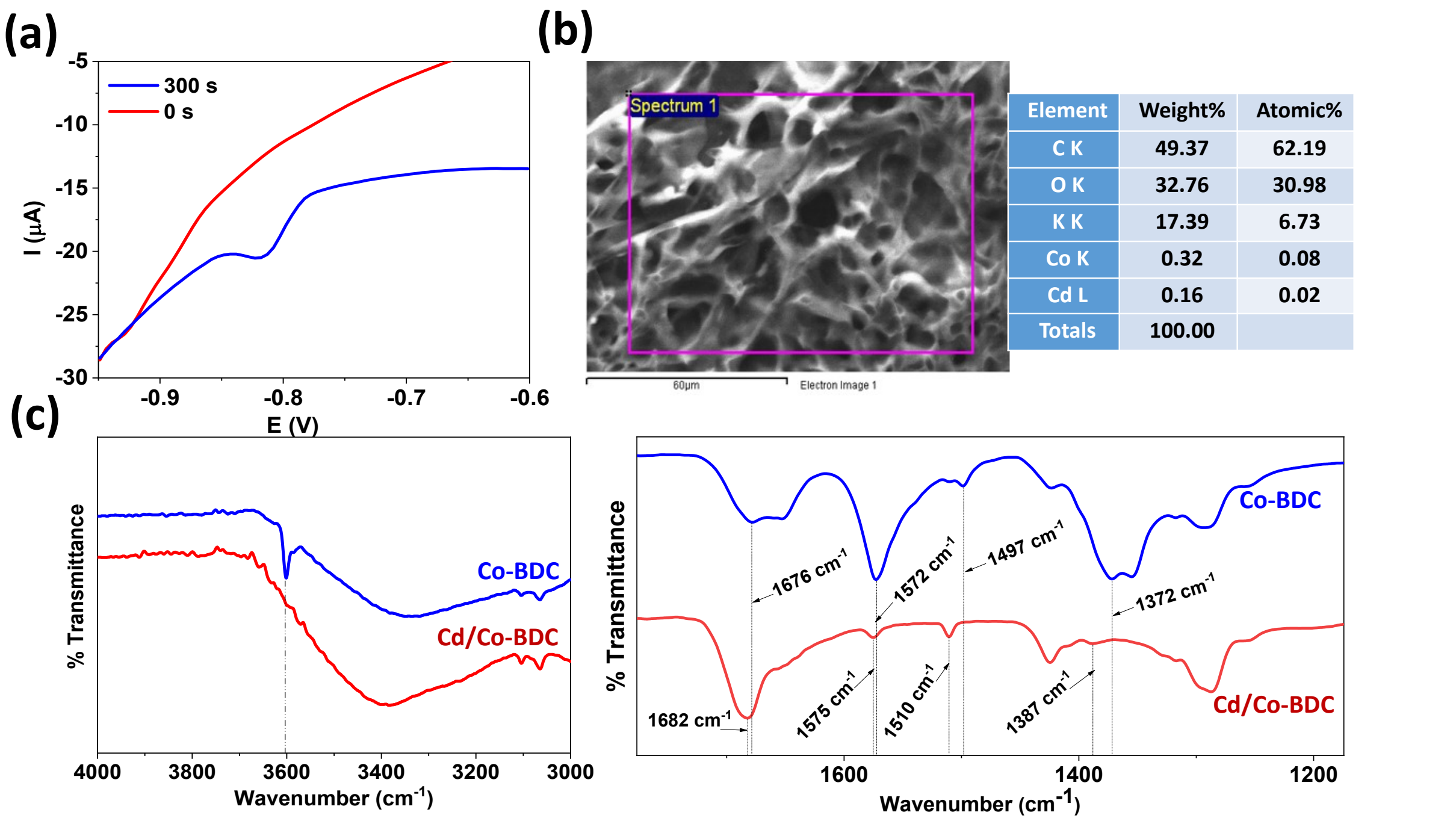

Fig. S6 (a) SWV reduction signals of Cd^{2+} recorded at the Co-BDC/GCE after 0 and 300 s adsorption at open-circuit potential in a 500 ppb Cd^{2+} solution. (b) EDX; and (c) Enlarged ATR-FTIR spectra showing selected wavenumber regions of the Co-BDC film before and after Cd^{2+} adsorption.

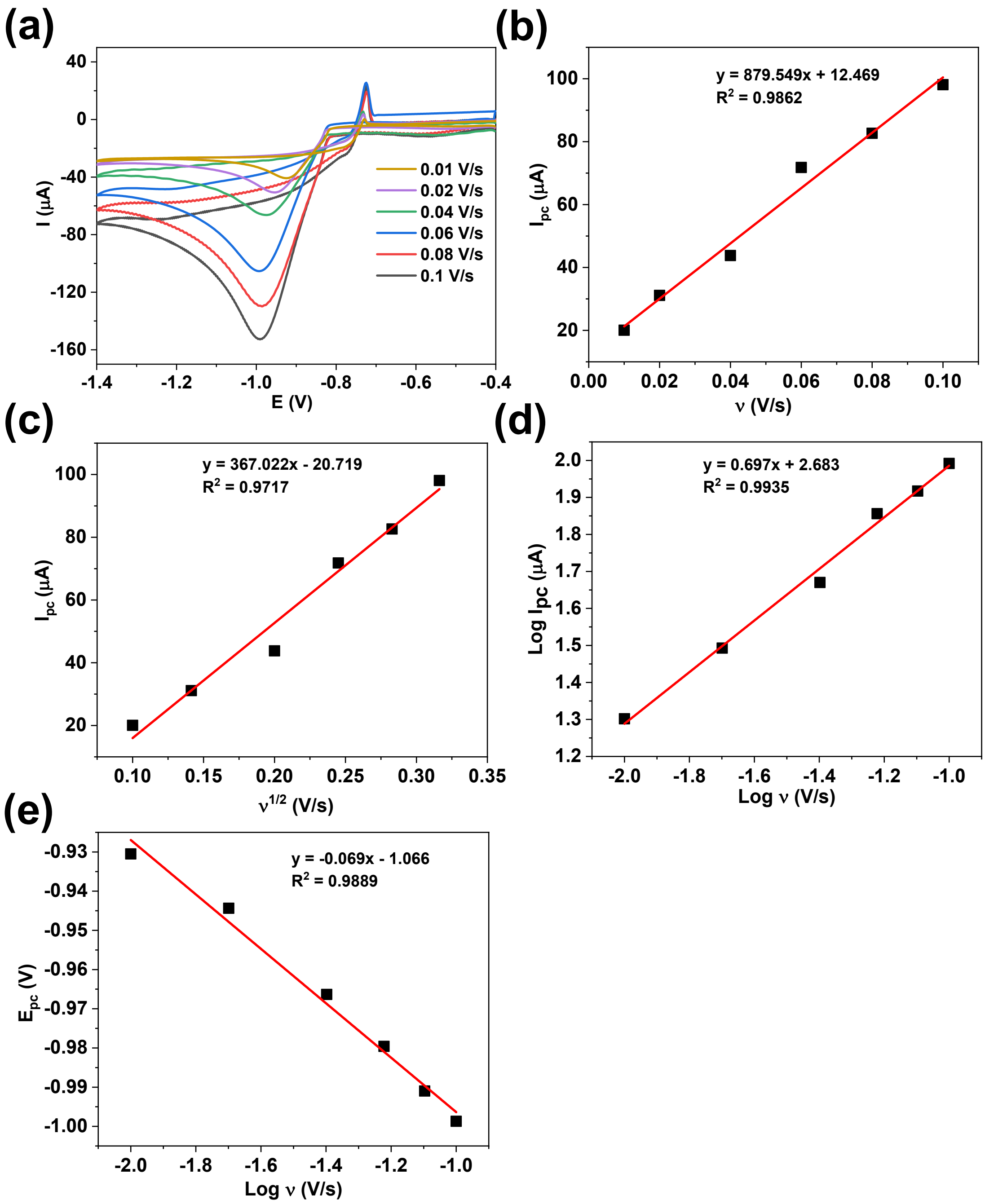

Fig. S7 Cyclic voltammograms of the Co-BDC/GCE in 0.1M KCl-HCl solution (pH 3.5) containing 2 ppm Cd^{2+} at different scan rates (a). Corresponding plots of (b) cathodic peak current (I_{pc}) versus scan rate (ν), (c) I_{pc} versus $ν^{1/2}$, (d) log I_{pc} versus log ν, and (e) peak potential (E_{pc}) versus log ν. The Co-BDC film was synthesized at -1.4 V for 300 s.

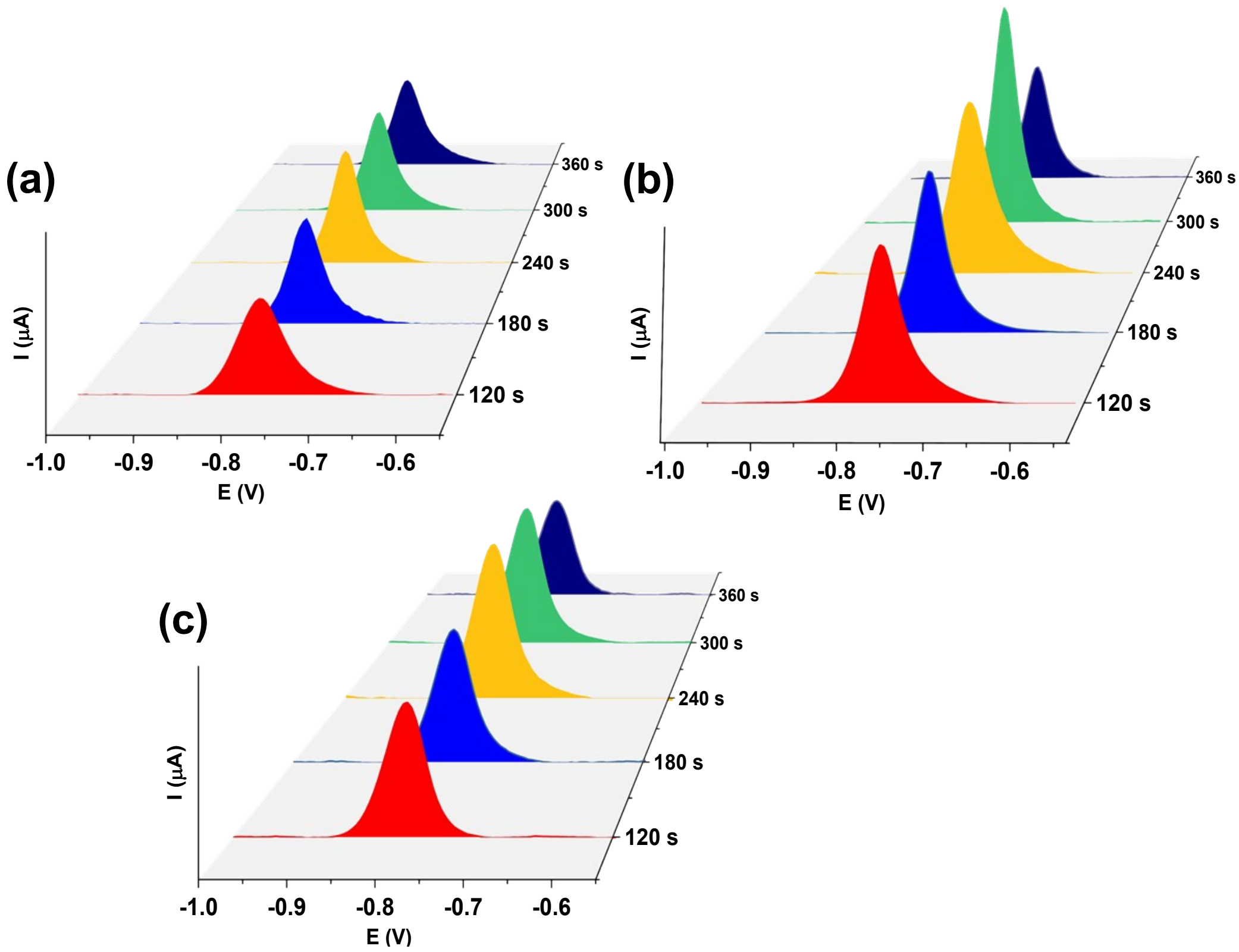

Fig. S8 SWV responses in 20 ppb Cd^{2+} solution for Co-BDC/GCE electrodes prepared at different deposition potentials (a) -1.2 V, (b) -1.4 V, and (c) -1.6 V with varying deposition times. Cd^{2+} signals were recorded in 0.1 M KCl-HCl (pH 3.5), at an accumulation potential of -1.1 V for 300 s.

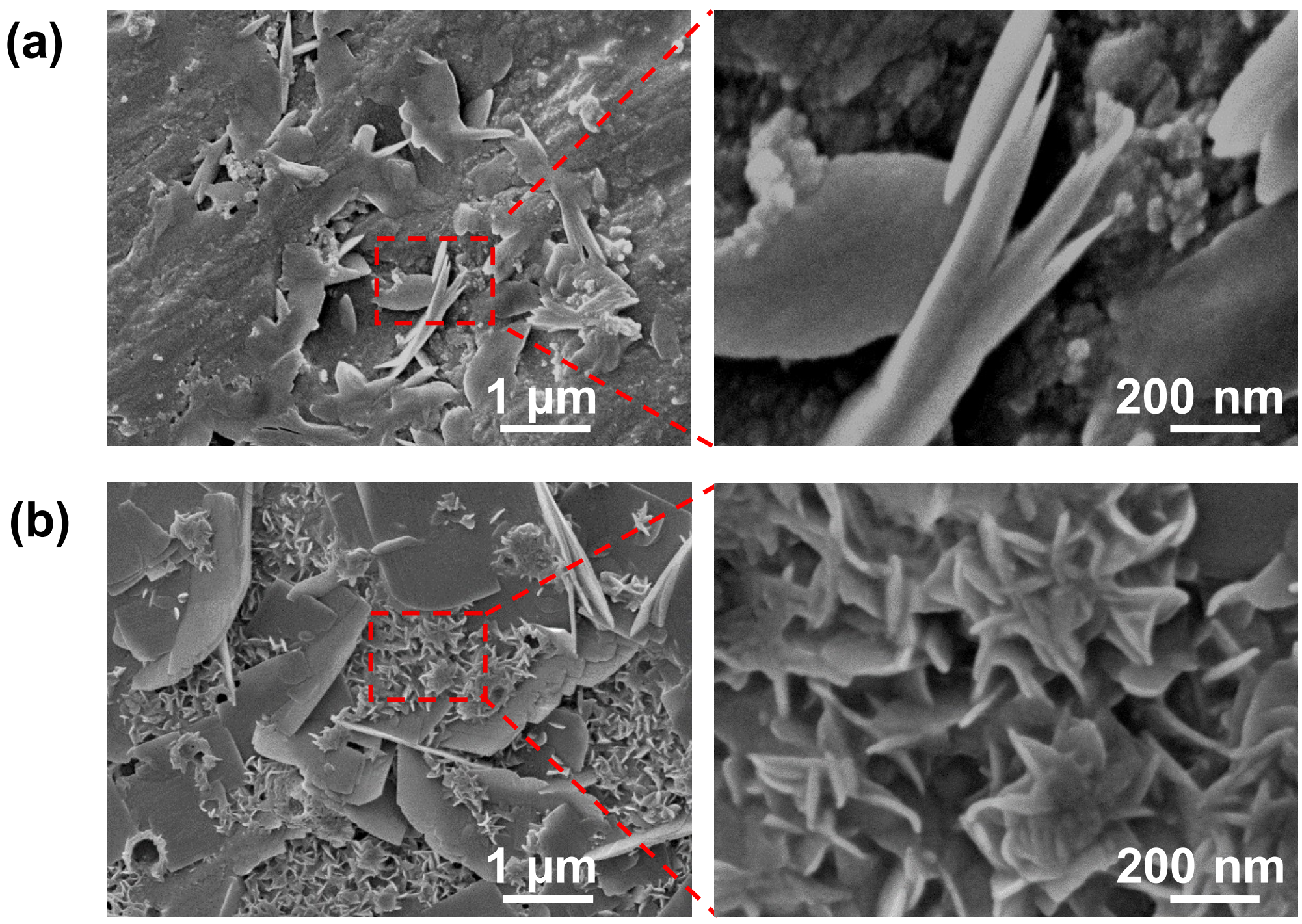

Fig. S9 FESEM images of Co-BDC/GCE electrodes synthesized at a deposition time of 300 s under different potentials: (a) -1.2 V and (b) -1.6 V.

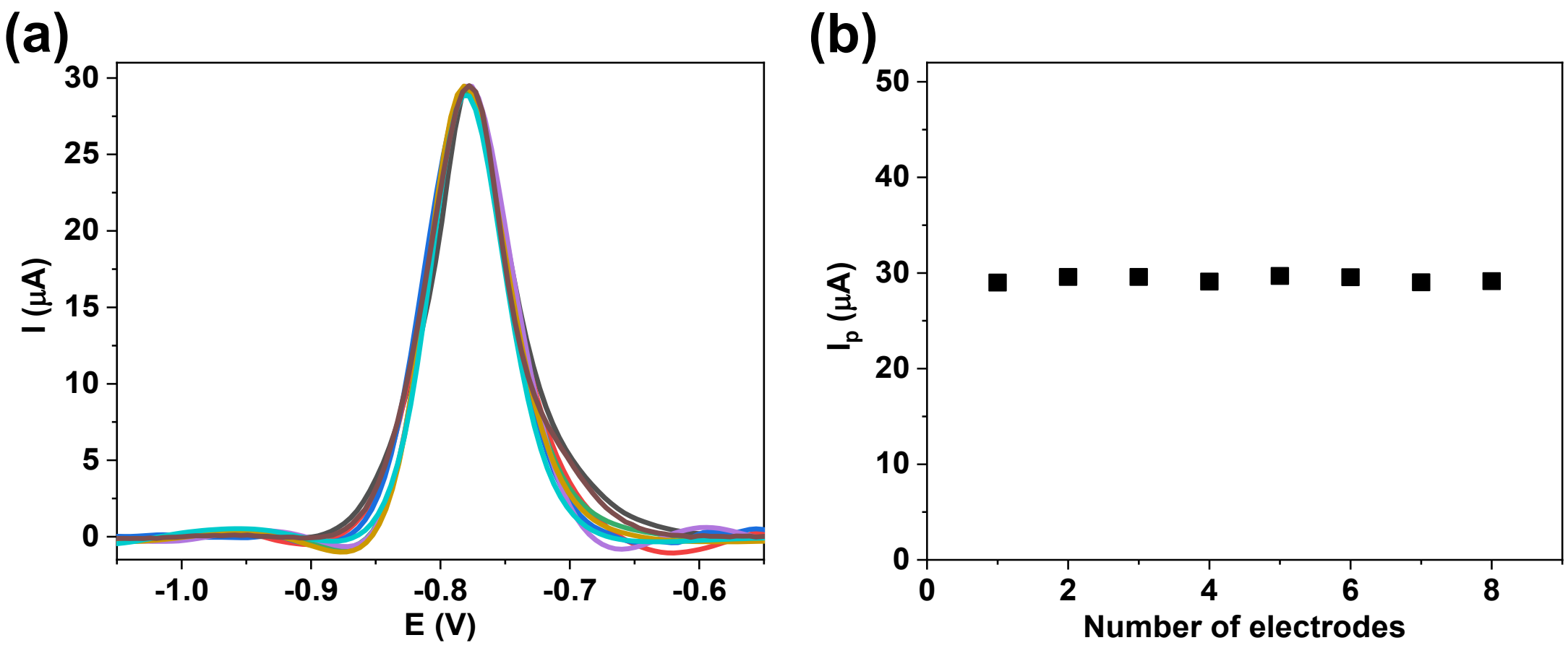

Fig. S10 (a) SWASV responses of eight independently fabricated Co-BDC/GCE electrodes in 20 ppb Cd^{2+} solution and (b) the corresponding Cd^{2+} peak heights. Measurements were performed in 0.1 M KCl-HCl solution (pH 3.5) at an accumulation potential of -1.1 V for 300 s.

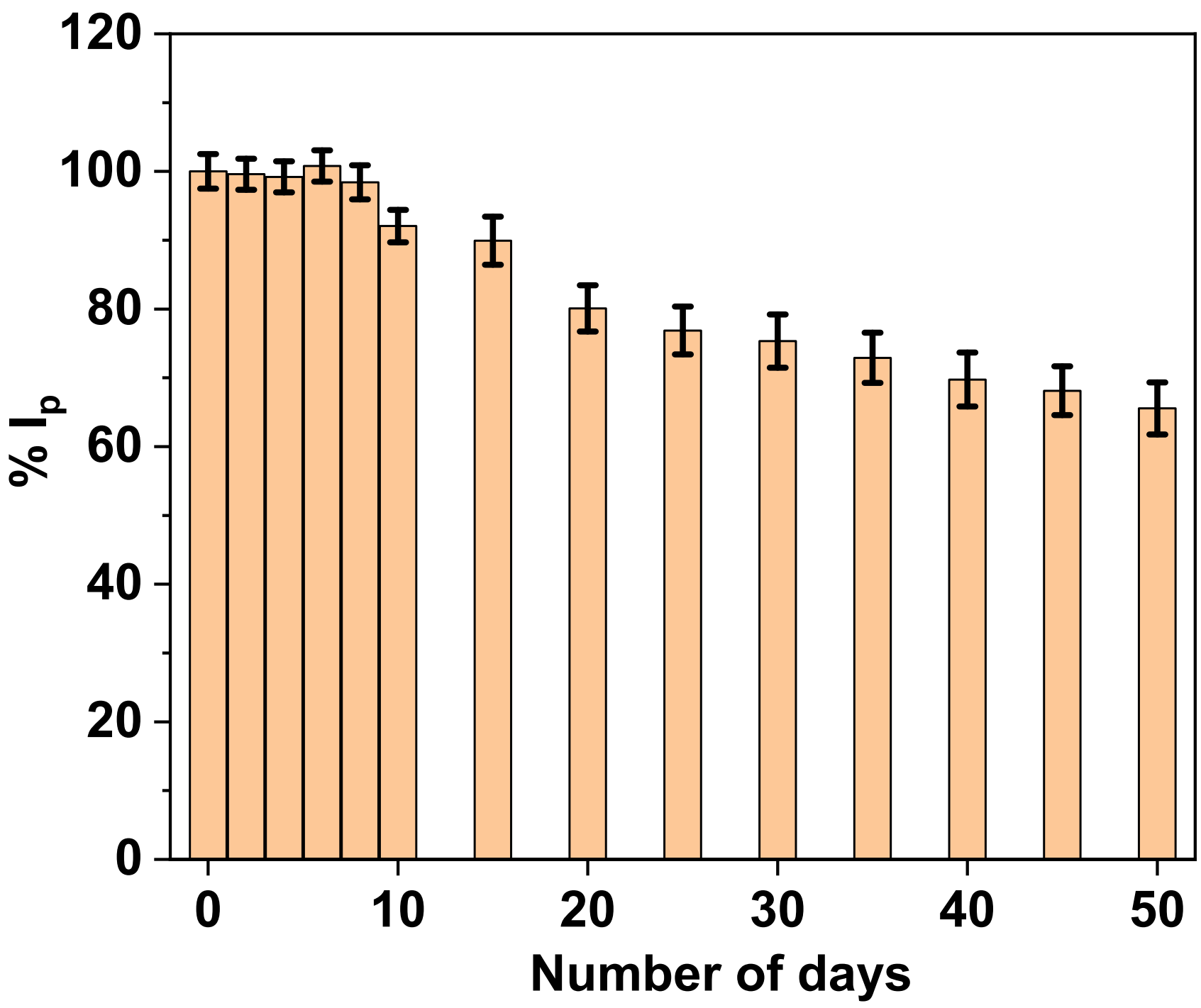

Fig. S11 Long-term stability of the Co-BDC/GCE evaluated by monitoring the response to 20 ppb Cd^{2+} after storage in a desiccator. Measurements were performed in 0.1 M KCl-HCl solution (pH 3.5) at an accumulation potential of -1.1 V for 300 s.

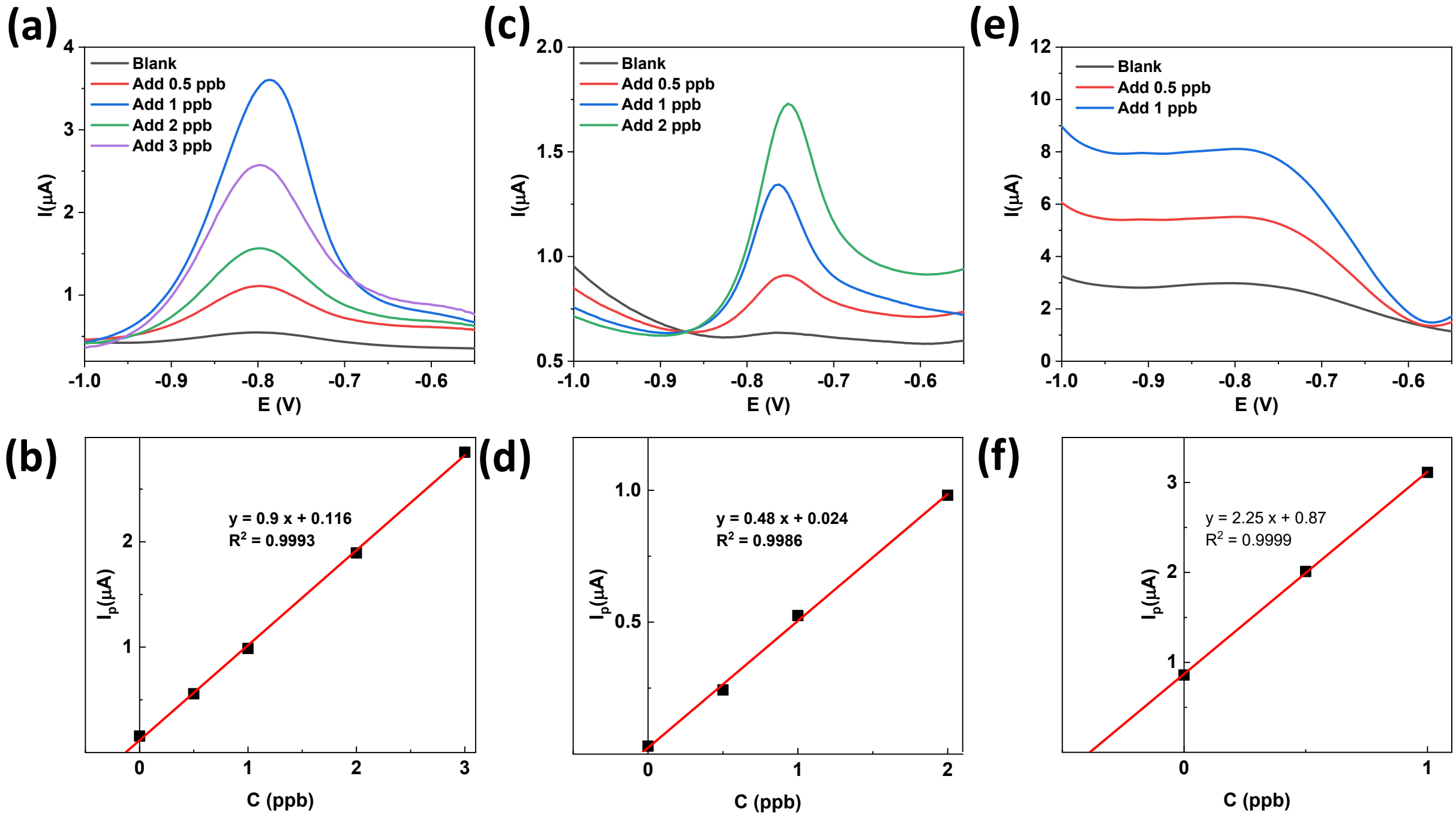

Fig. S12 SWASV voltammograms and the corresponding standard addition plots for Cd^{2+} determination in real samples using the Co-BDC/GCE: (a, b) West Lake water (Hanoi), (c, d) Cau River water (Thai Nguyen), and (e, f) sweet potato vegetable sample (Thai Nguyen).
